# Digital health interventions for non-communicable disease management in primary health care in low-and middle-income countries

**DOI:** 10.1038/s41746-023-00764-4

**Published:** 2023-02-01

**Authors:** Shangzhi Xiong, Hongsheng Lu, Nicholas Peoples, Ege K. Duman, Alberto Najarro, Zhao Ni, Enying Gong, Ruoyu Yin, Truls Ostbye, Lia M. Palileo-Villanueva, Rinchen Doma, Sweta Kafle, Maoyi Tian, Lijing L. Yan

**Affiliations:** 1 The George Institute for Global Health, Faulty of Medicine and Health, University of New South Wales, Sydney, NSW Australia; 2 Global Health Research Centre, Duke Kunshan University, Kunshan, China; 3 Wake Forest School of Medicine, Winston-Salem, NC USA; 4 Baylor College of Medicine, Houston, TX USA; 5 School of Anthropology and Museum Ethnography, Oxford University, Oxford, UK; 6 The Yenching Academy of Peking University, Beijing, China; 7 School of Nursing, Yale University, New Haven, CT USA; 8 School of Population Medicine and Public Health, China Academy of Medical Sciences & Peking Union Medical College, Beijing, China; 9 Department of Family Medicine and Primary Care, Lee Kong Chian School of Medicine, Nanyang Technological University, Singapore, Singapore; 10 College of Medicine, University of the Philippines Manila, Manila, Philippines; 11 Duke Global Health Institute, Duke University, Durham, NC USA; 12 School of Public Health, Harbin Medical University, Harbin, China; 13 Department of Preventive Medicine, Feinberg School of Medicine, Northwestern University, Chicago, IL USA; 14 The George Institute for Global Health, Beijing, China; 15 School of Health Sciences, Wuhan University, Wuhan, China

**Keywords:** Health services, Public health

## Abstract

Current evidence on digital health interventions is disproportionately concerned with high-income countries and hospital settings. This scoping review evaluates the extent of use and effectiveness of digital health interventions for non-communicable disease (NCD) management in primary healthcare settings of low- and middle-income countries (LMICs) and identifies factors influencing digital health interventions’ uptake. We use PubMed, Embase, and Web of Science search results from January 2010 to 2021. Of 8866 results, 52 met eligibility criteria (31 reviews, 21 trials). Benchmarked against World Health Organization’s digital health classifications, only 14 out of 28 digital health intervention categories are found, suggesting critical under-use and lagging innovation. Digital health interventions’ effectiveness vary across outcomes: clinical (mixed), behavioral (positively inclined), and service implementation outcomes (clear effectiveness). We further identify multiple factors influencing digital health intervention uptake, including political commitment, interactivity, user-centered design, and integration with existing systems, which points to future research and practices to invigorate digital health interventions for NCD management in primary health care of LMICs.

## Introduction

Digital health interventions—known as “a discrete functionality of digital technology that is applied to achieve health objectives”—have exceptional potential to promote universal health coverage and enhance health service delivery^1,2^. In May 2018, the World Health Assembly passed the Digital Health Resolution, recognizing the potential of digital technologies to support health systems by improving the accountability, availability, accessibility, continuity, utilization, and effectiveness of health care^3^. The World Health Organization (WHO) further classified digital health interventions according to four types of users, including 28 categories and 87 sub-categories^4^. These users, categories, and sub-categories of digital health interventions cover various areas of health systems with a particular focus on health service delivery. One area that has great potential for improvements through digital health interventions is the management of non-communicable disease (NCD) in primary health care.

Distinct from hospital-level specialist care, primary health care emphasizes first-contact, accessible, continued, comprehensive, and coordinated patient-focused care, and is often the closest to where people live^5^. Primary health care has been recognized as the cornerstone of combating NCDs worldwide^6,7^. This is because NCDs—such as hypertension, diabetes, and cardiovascular diseases—are characterized by long disease durations and a continuous need to anticipate and mitigate risk factors through lifestyle modifications^8–11^, which is better addressed by primary health care than higher-level health facilities. The literature, however, has shown that substantial gaps exist in most primary healthcare systems, particularly in low-and middle-income countries (LMICs), including limited human resources and capacity, shortages in medicines and equipment, and suboptimal quality of care^11–13^. These constraints prevent primary healthcare facilities from achieving optimal NCD management.

In the past decade, many studies have explored whether and how digital health interventions can contribute to bridging such gaps. The World Heart Federation recently released a roadmap for digital cardiology, where it was acknowledged that digital health interventions had potential to help address health system challenges and achieve optimal and universal health coverage by promoting health service coverage, empowering patients and providers, and improving long-term outcomes^14^. The digital cardiology roadmap considered a diversity of digital health interventions, spanning text messaging, telehealth, and electronic decision support tools^14^. The CONNECT trial conducted in Australia, for example, used an interactive web-based app linked to the electronic health records (EHRs) in primary care, and found borderline improvements in blood pressure and lipids control, and significant effectiveness in increasing physical activity^15^. The TEXTME trial found sending text messages to people with heart disease was associated with improvements in blood pressure control, diet, physical activity, and smoking reduction^16^. Other existing reviews found text messages to be of low costs and effective in addressing modifiable NCD risk factors such as medication compliance^17^, and weight management^18^. However, most of these original studies and reviews focused on high-income countries, hospital settings, or fields other than NCDs^15,16,19–22^. The literature on digital health interventions for NCD management in primary healthcare settings and LMICs are fragmented and sparse.

This scoping review aims to synthesize evidence on the current use of digital health interventions for NCD management in the primary health care of LMICs. Specific objectives include: (1) to identify gaps in the use of digital health interventions for NCD management in primary health care of LMICs by benchmarking existing studies with the WHO digital health classification; (2) to explore the effectiveness of existing digital health interventions by different outcomes; and (3) to identify factors influencing the uptake of these digital health interventions through narrative synthesis.

## Results

### Search results

**Fig. 1:**
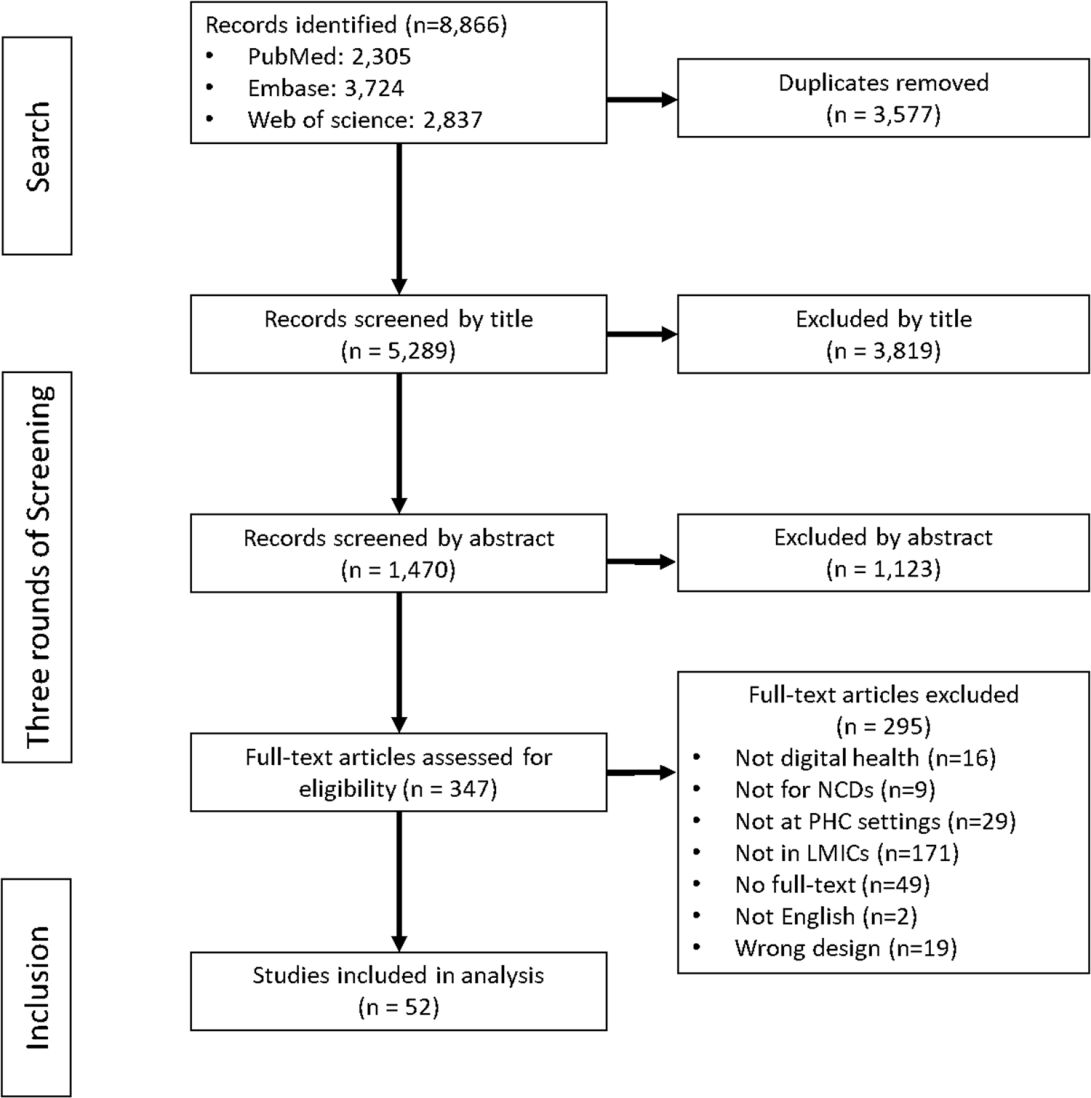
Flowchart for study search and screening. The initial results were 8866 items. After duplicate removal and screening by title, abstract, and full-text, a total of 52 papers were included for final analysis.

We identified a total of 8866 records in the search from the three English databases, and 3577 duplicates were removed across the databases (Fig. 1). After screening by title and abstract, 347 items remained for further screening. In the third round of screening by full-text, 295 items were further excluded. The primary reasons for this round of exclusion included: “not conducted in LMICs” (*n* = 171), “no available full-texts” (e.g. conference abstracts, *n* = 49), and “not conducted at primary health care settings” (*n* = 29). A total of 52 papers were included for final analysis.

### Study characteristics

There were 31 reviews and 21 trials among the final included studies (see Supplementary Tables 2 and 3 for data extraction tables on basic characteristics of included reviews and trials, respectively). There were three major review types in the 31 review papers:^23–53^ systematic reviews (*n* = 22), scoping reviews (*n* = 3), and umbrella reviews (*n* = 4), in addition to two non-specified types of literature reviews, one of which reviewed mobile phone APPs rather than papers^38^. Among the 22 systematic reviews, six conducted meta-analysis, and most of the other papers mainly adopted narrative synthesis. Various types of publication were covered in these review studies, including randomized controlled trials (RCTs), cluster RCTs, quasi-experiments, pre-post experiments, observational studies, and literature reviews.

The 21 trials were conducted in various LMICs^54–74^, including eight from Brazil, three from Thailand, three from India, two from China, and one from Kenya, Chile, Malaysia, South Africa, Turkey, and Argentina, separately. Of note, one of the trials was conducted in both China and India^71^. Regarding trial designs, there were four RCTs, five cluster RCTs, seven quasi-experiment studies, and five feasibility/pilot studies. Most of these trials had either people with NCDs or primary healthcare providers as participants, and three studies had both. There was a wide range of sample sizes (from 10 to 6979) across these trials, reflecting high heterogeneity in study designs. The most common type of participants in the trials are those with hypertension or diabetes.

For quality assessment, more than half of the included trials were of suboptimal quality (*n* = 12), with eight of them of good quality and one with fair quality. The most common factors that compromised the studies’ quality were the lack of randomization (*n* = 11), lack of evidence on sample size sufficiency (*n* = 10) and absence of pre-specification for study outcomes (*n* = 7), which was mainly due to the high proportions of quasi-experiment and feasibility/pilot studies.

### Digital health interventions in selected studies

We identified a total of 11 types of digital health interventions for NCD management in primary health care from the selected studies, which covered 14 out of 28 categories based on WHO digital health classifications (Table 1). Eight of the identified digital health interventions were used by primary healthcare providers, such as EHR, decisions support systems, and telemonitoring devices. Five interventions were used by healthcare clients (i.e. people with NCDs), including short messaging services (SMS), multimedia message services, and interactive voice responses or phone calls. Two were used by both healthcare providers and clients: web-based/online telecare platforms, and smartphone applications. Of note, EHR can also be classified as being used for “data services”, the fourth type of user according to the WHO classification, given the nature of EHR being collecting routine health and medical information of people using health services. We did not find any digital health interventions that were used by health system managers from the selected studies.

**Table 1:** Identified digital health interventions for non-communicable disease management in primary health care.

Digital health interventions	WHO classification of digital health interventions	WHO quality of care dimensions	Study numbers in references
For clients (*n* = 5)			
Short message services	Targeted client communication	Accessibility, acceptability	Reviews:^27–30,32,34–36,39,42,46–52^ Trials:^27,56,61,72^
Multimedia message services	Targeted client communication	Accessibility, acceptability	Reviews:^28,30,32,35,36,39^
Interactive voice response or phone calls	Targeted client communication	Accessibility, acceptability	Reviews:^24,27,34–36,42,44,45,47,50^ Trials:^56,60,68^
Web-based/online telecare platforms	Targeted client communication, untargeted client communication, on-demand information services to clients	Efficiency, accessibility, acceptability, equity	Reviews:^24–26,34,35,39,42,47^ Trials:^59^
Smartphone applications	Targeted client communication, untargeted client communication, personal health tracking, on-demand information services to clients, client-to-client communication	Effectiveness, efficiency, accessibility, acceptability, equity	Reviews:^27,28,30,33–38,42,45–51,53^ Trials:^57,64,65,74^
For healthcare providers (*n* = 8)			
Electronic health/medical record	Client health records, client identification and registration	Effectiveness, efficiency	Reviews:^27,28,34,41^ Trial:^74^
Decisions support systems	Healthcare provider decision support	Effectiveness, safety	Reviews:^34,40,43^ Trials:^54,62,64,69–71^
Digital-based provider training sessions	Healthcare provider training	Effectiveness, accessibility, equity	Reviews:^38,40,49^ Trials:^58,59,74^
Telemonitoring devices, including point-of-care systems	Telemedicine, laboratory and diagnostics Imaging management	Effectiveness, efficiency, accessibility, safety	Reviews:^23,25,26,31,35,37,41,51^ Trials:^60,63,66,73^
Digital-based health examination report, screening, and diagnosis	Laboratory and diagnostics imaging management	Effectiveness, efficiency, safety	Trials:^62,67^
Electronic prescriptions	Prescription and medication management	Effectiveness, efficiency, safety	Reviews:^26^
Web-based/online telecare platforms	Telemedicine	Efficiency, accessibility, equity	Reviews:^25,26,34,35,39,47^ Trials:^59^
Smartphone applications	Targeted client communication, untargeted client communication, healthcare provider communication, telemedicine, client health records, healthcare provider decision support	Effectiveness, efficiency, accessibility, acceptability, equity	Reviews:^27,28,30,34–38,45,46,48–51,53^ Trials:^64,65,70,71,74^
For data services (*n* = 1)			
Electronic health/medical record	Data collection, management, and use	Effectiveness, efficiency	Reviews:^27,28,41^

Benchmarked against World Health Organization’s digital health classifications, 14 out of 28 digital health intervention categories were found, and most of them focused on improving health service efficiency and accessibility.

From the perspective of the WHO digital health intervention classifications, the majority of these interventions were used for communications (Table 1), including targeted client communication (*n* = 6), untargeted client communication (*n* = 3), healthcare provider communication (*n* = 1), and client-to- client communication (*n* = 1). Other major types of digital health interventions were about patient information, including client health records (*n* = 2), on-demand information services to clients (*n* = 2), and personal health tracking (*n* = 1). Other classified digital health interventions included telemedicine (*n* = 3), laboratory and diagnostics imaging management (*n* = 2), healthcare provider decision support (*n* = 2), healthcare provider training (*n* = 1), client identification and registration (*n* = 1), and prescription and medication management (*n* = 1). Compared with the WHO classification, there were major under-use of digital health interventions in these studies. For example, there was no intervention in “citizen-based reporting”, “client financial transactions”, “referral coordination”, and “health worker activity planning and scheduling”, and there were no digital health interventions for healthcare managers, such as “human resource management” and “supply chain management”. Regarding data services, the current intervention (i.e., EHR) was only limited to basic “data collection, management, and use”, and there were no reported interventions about “data coding”, “location mapping”, or “data exchange and interoperability” in the selected studies.

From the perspective of WHO quality of care dimensions, most of these digital health interventions focused on improving health service efficiency (*n* = 9) and accessibility (*n* = 9), followed by effectiveness (*n* = 9), while acceptability (*n* = 6), equity (*n* = 5), and safety (*n* = 4) issues were relatively less addressed in the selected studies (Table 1).

### Effectiveness of digital health interventions

**Fig. 2:**
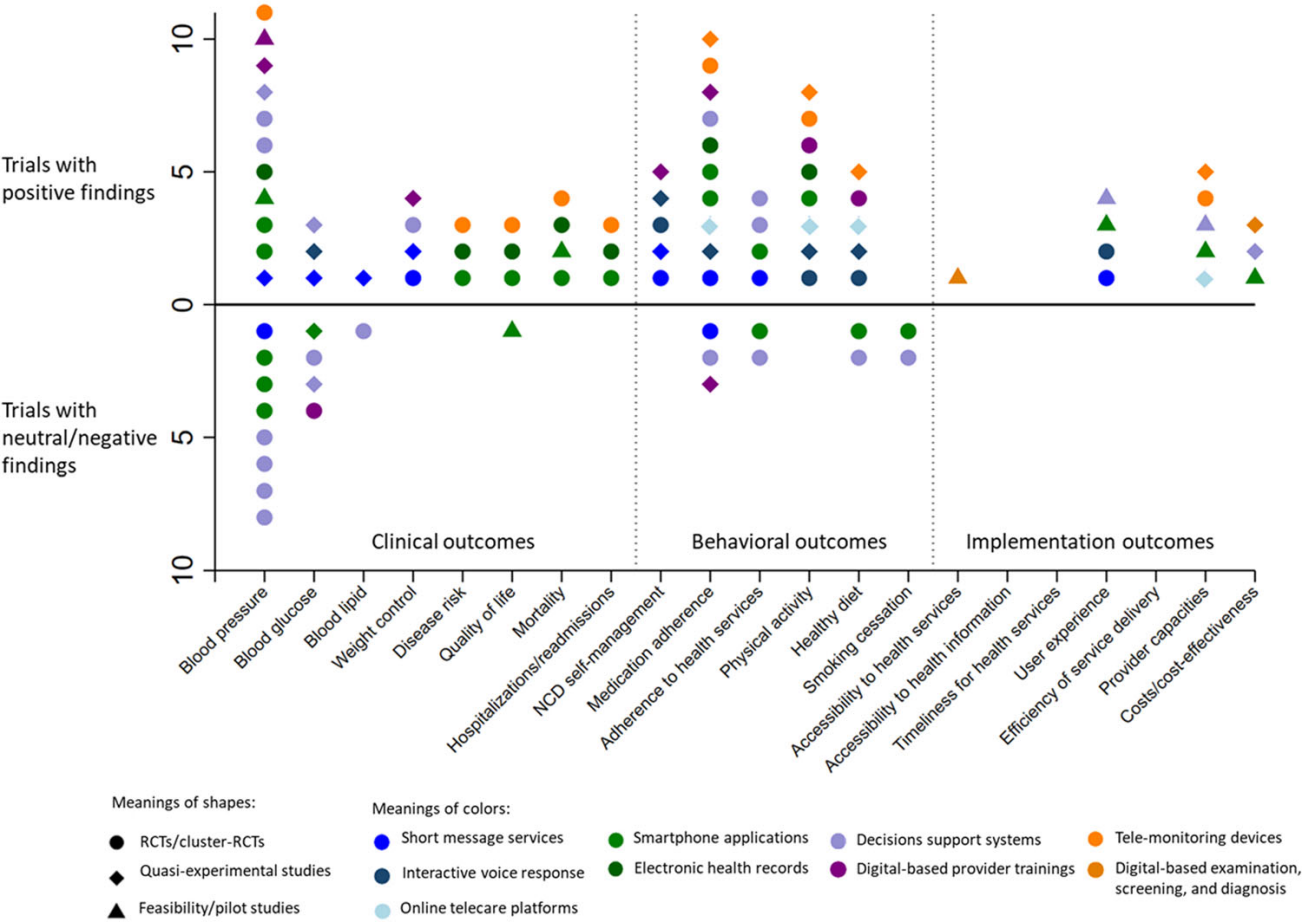
The effectiveness of digital health for non-communicable disease management in primary health care in included trials. We found three major types of outcomes from all included trials, and digital health interventions’ effectiveness varied among clinical (mixed), behavioral (positively inclined), and service implementation outcomes (clear effectiveness).

**Fig. 3:**
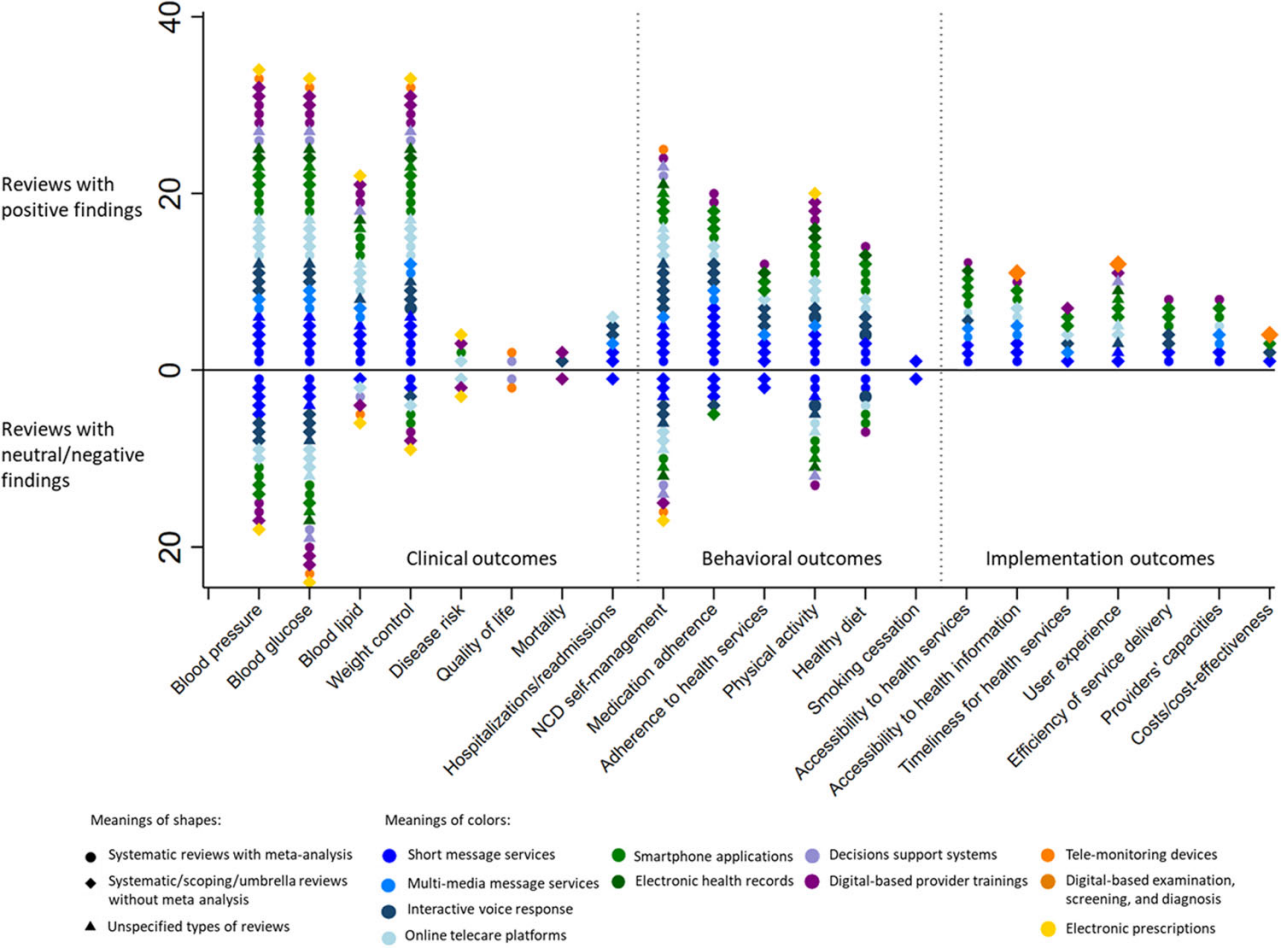
The effectiveness of digital health for non-communicable disease management in primary health care in included reviews. We found three major types of outcomes from all included reviews, and digital health interventions’ effectiveness varied among clinical (mixed), behavioral (positively inclined), and service implementation outcomes (clear effectiveness).

The included studies presented a variety of digital health interventions that intended to address a wide range of outcomes, making it unfeasible to conduct rigorous quantitative synthesis such as meta-analysis. Therefore, we explored the effectiveness of digital health interventions by documenting the positive and negative/neutral findings from different of outcomes (Figs. 2 and 3). We found three major types of outcomes from all included studies: (1) eight types of clinical outcomes for individuals including control for blood pressure, blood glucose, hospitalization, and mortality; (2) six types of behavioral outcomes for individuals, including self-management activities such as medication adherence, and health behaviors such as diet and physical activity; and (3) seven types of implementation outcomes for health services, which refers to factors associated with the process of health service provisions, such as service accessibility, user experience, and primary healthcare providers’ capacities.

In general, the effectiveness of digital health interventions differed across these three types of outcomes (Figs. 2 and 3). First, the findings for clinical outcomes, such as blood pressure and blood glucose control, were highly mixed, where both positive and negative/neutral results were common in both trials and reviews. Many of the included review papers found mixed findings within their own identified studies^23,31,40,45,51,52^. Second, the effects of digital health interventions on individual behavioral outcomes were more positively inclined, where many studies showed a significantly positive impact on disease self-management activities and healthy lifestyles, such as improved adherence to medicines, adherence to health services, and physical activity. Some of the studies, although less prevalent, also found non-significant results on behavioral outcomes. Third, for health service implementation outcomes, results were consistently positive among both trial reviews, where digital health interventions improved the accessibility and user experience in health service delivery, improved primary healthcare providers’ capacities, and/or with better cost-effectiveness. Notably, several papers suggested the lack of rigorous cost-effectiveness analysis in the current digital health studies^25,29,47^.

Several patterns were noticeable and informative regarding the effectiveness of different types of digital health interventions. First, the communication-related digital health interventions (i.e., SMS, multimedia message services, and online platforms, shown in different shades of blue) were the most widely explored in the included studies. Among the trials (Fig. 2), several quasi-experimental studies documented the positive effects from these interventions in the control of blood pressure, glucose, lipids, and weight, and many RCTs also found their effectiveness in improving people’s adherence to medication and health services. However, in the reviews (Fig. 3), the effects of these interventions were highly mixed in all types of outcomes except for implementation outcomes. Second, both the trials and reviews suggested that digital health interventions that aimed to strengthen providers’ capacities (i.e., decision support systems and online trainings, shown in different shades of purple) were substantially mixed in almost all the clinical and behavioral outcomes. Third, the digital-based clinical practices (i.e., screening, diagnosis, prescribing, and monitoring, shown in different shades of orange) were found to be consistently effective in all types of outcomes in the trials, but in the reviews their effectiveness in clinical outcomes were mixed.

### Factors influencing digital health uptake

Based on our inductive content analysis on the included studies, we identified multiple factors influencing the uptake of digital health interventions for NCD management in primary health care of LMICs (Table 2). Based on the nature of different stakeholders, we further classified these factors into four groups: (1) factors regarding policymakers (*n* = 2), (2) factors regarding technological industry (*n* = 4), (3) factors regarding digital health designers (*n* = 7), and (4) factors regarding digital health users (*n* = 6).

**Table 2:** Factors influencing real-world uptake of digital health interventions for non-communicable disease management in primary health care.

Factors influencing digital health uptake	Study numbers in references
Factors regarding policymakers	
Political commitment is key in digital health innovation and uptake, through advocate, financial support, and stakeholder engagement.	Reviews:^38,41,47,49^
Lack of political commitment, including regulations, standardization, monitoring, and evaluation, could be important barriers to digital health uptake.	Reviews:^38,49^
Factors regarding the technological industry	
The development in technologies and infrastructures provide solid ground for digital health advancement, including penetration of cell phones and the internet.	Reviews:^23,24,26,28,30,38,41,44,47,49,51,55^ Trials:^55,56,58,63^
Technical constraints (e.g., cell phone unavailability, internet instability) in marginal and remote regions limit the coverage of digital health.	Reviews:^34,44,47,49^ Trials:^65,68,70^
Some unresolved data-related technical issues prohibit further uptake of digital health, including data security issues and data ownership issues.	Reviews:^33,41,46,47,50,55^
Lack of interoperability across different digital health systems is an important barrier for integration, especially with EHR, APPs, and online platforms.	Review:^41^
Factors regarding digital health designers	
Digital health tools that enable two-way human interactions are better accepted while replacing human communications with automatic responses is less favored.	Reviews:^26,32,34,39,41,52^
Incorporating behavioral theories in digital health design is a facilitator, and the absence of that could be a barrier.	Reviews:^29,39,52^
Having user-centered design, such as involving target users in the design phase of digital health could be a facilitator (i.e., co-design).	Reviews:^31,46,50^ Trial:^71^
Tailored design and personalized contents for patients is a facilitator, and one-for-all unified contents and designs could be a barrier.	Reviews:^34,39,50,52^
Incorporating users’ feedback on acceptability and satisfaction is a facilitator, and lack of that could be a barrier and is currently under-addressed.	Reviews:^33,43^
Digital health designs that were integrated into existing healthcare model was a facilitator for successful uptake, and unintegrated digital health interventions that impose additional workload for primary healthcare providers is a barrier.	Review:^24^ Trials:^70,71,74^
Easily navigable interface is a facilitator, and poor/complicated interface is a barrier for the uptake of digital health tools.	Reviews:^34,40,47^ Trials:^65,66,68^
Factors regarding digital health users	
Adequate high-quality personnel training for primary healthcare providers is a facilitator for digital health uptake, while lack of that could be a barrier.	Reviews:^40,53,55^ Trials:^69^
Lack of local capacities, such as technological illiteracy, are barriers to digital health uptake.	Reviews:^52,55^ Trials:^63,65,68^
Lack of guidance for patients is subject to low acceptance and waning interests in using digital health over time.	Reviews:^26,32,33,40,51^ Trial:^57^
Designing incentives/motivations for primary healthcare providers and patients to use digital health facilitates the uptake, while lack of that could be a barrier.	Reviews:^34,45,51,55^ Trials:^59,68,70^
Suboptimal quality and accuracy for data input, report, and interpretation is a barrier to effective use of digital health.	Reviews:^32,41,44^ Trials:^66,70^
The mobilization of local communities with local capacity improvements is a facilitator to enhance the use of digital health tools, and lack of those could be a barrier.	Review:^43^ Trials:^59,70^

Multiple factors influenced the real-world uptake of digital health interventions, including factors regarding policymakers, factors regarding technological industry, factors regarding digital health designers, and factors regarding digital health users.

First, political commitment with regulations that encourage and standardize the use of digital health tools was reported as strong facilitators, while the absence of that in many settings could be barriers. Second, for the technology industry, a frequently mentioned factor that facilitated the uptake of digital health was the technological advancement and the prevalent use of information and communication technologies, such as the wide penetration of cell phones and the internet. Some less-developed places and populations such as in rural regions, however, were still faced with technical constraints and limitations. Moreover, lack of interoperability across different digital health platforms, unresolved data security, and ownership issues are barriers to further uptake of digital health interventions (Table 2).

The design of digital health interventions was also considered to influence their uptake (Table 2). Major factors that contributed to optimal designs included the incorporation of existing behavioral science theories, tailored personalization as opposed to one-for-all contents, the emphasis on human interactivity, the consideration of users’ feedback, and the involvement of digital health target users in the design phase (i.e., co-design). Finally, from the perspective of digital health users, adequate training for primary healthcare providers and guidance for people with NCDs on using digital health tools and customized incentives and motivations for sustainable use of digital health tools were reported as important factors that facilitated the uptake of digital health interventions. Lack of local capacity, on the other hand, such as technological illiteracy and suboptimal quality of data input and report, were reported as barriers to the uptake of digital health.

## Discussion

This study provides a holistic review on digital health interventions for NCD management in primary health care of LMICs for the past decade. We found 52 relevant studies and identified 11 digital health interventions mainly used by two types of users: primary healthcare providers and people with NCDs. This suggested the under-use of digital health interventions compared with WHO recommendations^3,4^. We found the effectiveness of digital health interventions to be highly mixed for clinical outcomes, more positively inclined for behavioral outcomes, and consistently promising for service implementation outcomes. We also identified many factors that influenced the uptake of digital health interventions from policy maker, technical industry, designer, and user perspectives. Amidst the substantial and increasing global burden of NCDs^11^, our synthesis of evidence on digital health may shed light on further exploration of digital enhancements to health systems, particularly in primary health care of LMICs.

The paucity of digital health interventions applied in NCD management in primary health care of LMICs is disproportionate to the high demand for health system strengthening. A recent rapid scoping review found that the vast majority of digital health interventions for NCD management during the COVID-19 pandemic were conducted in high-income countries and were mostly hospital-based^75^. An earlier study identified 12 common domains of mobile health interventions primarily used for maternal and child health, which covered almost all the digital health interventions identified in our study and more, such as human resource and supply chain management^76^. The majority of the digital health interventions identified in our study, however, was focused on “communications”, such as SMS and smartphone applications, aiming to improve health service efficiency and accessibility. Such digital health interventions do not require additional equipment or infrastructures except for cell phones and internet, which is already widely penetrated in the general populations and thus poses minimum additional costs. In contrast, we found very limited use for digital health interventions that entailed infrastructure updates and systemic enhancements, such as those for data collection, management, and analysis to support health administration. This is in line with findings from another review of 207 published studies, which identified major gaps in the infrastructure and information systems in the primary healthcare systems of LMICs^12^. On the other hand, regions with higher standards of digital health have been exploring and practicing the use of massive and dynamic NCD data to inform public health governance and policymaking^77^. Enabled by well-developed digital health data services, researchers have also been using EHR systems to support the conduct of clinical trials^78^. These aspirations warranted substantive input to enhancing the infrastructure of primary healthcare systems in LMICs, particularly to strengthen the health information systems.

Besides infrastructure constraints, another potential explanation for the under-use of digital health interventions in primary health care of LMICs is lack of local innovation. Evidence shows the best solutions are those that are responsive to local contexts^16,79^. However, current digital health interventions in LMICs relied on importing existing technologies from other settings to places they were not created for, sometimes in one-off research projects. A review of eHealth interventions in Nepal, for example, found that many were not adequately integrated into the existing health systems^80^. This limitation compromised the appropriateness, efficacy, and scale-ability of digital health interventions in LMICs. From the perspective of innovation diffusion^81^, the use of digital health interventions for NCD management in primary health care of LMICs could be classified as “late majority” or even “laggards” in some regions (as opposed to “innovators”, “early adopters”, and “the early majority”). This may also partially explain our mixed findings on the effectiveness of digital health interventions in LMICs, and warrants further attention to not only translate digital health interventions from high-income countries but also to encourage digital health innovations that derive from local contexts and needs.

Despite the “laggardness” of digital health interventions, the included studies still presented high heterogeneity in the outcomes of using digital health, which was also found in other systematic reviews^19–21^. Such commonly acknowledged heterogeneity in digital health designs and outcome selection is informative in itself—it signals a diversity of possibilities for the role of digital health interventions in health care. However, this could also imply that digital health as an emerging field might be too broad and inclusive a concept to draw definitive conclusions at least for now, especially at settings where rigorous evidence is sparse. It might be more plausible for further research to develop more granular foci on fractions of digital health with shared homogeneity.

Therefore, to mitigate concerns of such heterogeneity, our study further categorized the various types of study outcomes as either clinical, behavioral, or implementation outcomes. Among these three types of outcomes, the effectiveness of digital health varied greatly in the levels of consistency, if not direction. With respect to clinical outcomes of individuals, some studies attributed the highly mixed and neutral results to the study design limitations, such as small sample size and short follow-up durations^40,52^. This mixture of findings on clinical outcomes added to the conflicting evidence in the existing literature, where many meta-analyses of related topics disagreed on outcomes such as blood pressure reduction^19,20^. This disagreement may result from differences in study locations (high-income countries or LMICs), populations (general population or less advantaged populations), and study settings (hospitals or primary health care)^19,20^.

For individuals’ behavioral outcomes, although still mixed, we consistently observed positively inclined results, especially when substantiated by behavioral science theories^26,82^. Existing studies on NCD management listed four major NCD behavioral risk factors—tobacco use, harmful alcohol consumption, unhealthy diet, and physical inactivity^11^. We found the latter two had widely documented positive improvements in our included studies. We also found positive improvements in disease management activities, such as adherence to medications and regimens, consistent with the existing literature^20,21,83^. Nevertheless, the long-term sustainable effects of digital health on behavioral changes remains uncertain^19,20,84^. Further digital health evidence needs to be not only scaled up in scope but also in the duration of observation.

For health service implementation outcomes, we consistently found positive effects from digital health interventions. This included improved user experience, timeliness of and accessibility to health care and information, consistent with studies in other settings^20,85,86^. Although this is promising, the WHO’s guideline on digital interventions expected even more on health system strengthening^3^, including improving health service coverage, service awareness and utilization, availability and capacity of human resources, availability of commodities and equipment, and service continuity and effectiveness. Following these aspirations, instead of treating health service implementation outcomes mainly as secondary outcomes or process indicators, as did in most of the included studies, a better way forward may be to focus on them more, which could produce more tangible and reliable evidence that is also greatly needed.

Based on our scoping review, it could be safely inferred that digital health interventions are essentially an empowerment strategy for individuals’ health and disease management and for health facilities’ service delivery. In practice, however, effects are ultimately subject to digital health interventions’ real-world uptake, which is influenced by many factors. A study on digital interventions for mental health mentioned an “enormous research-to-practice gap” between 15 years of evidence from efficacy trials and “virtually no successful and sustainable implementation” of digital health in real world^22^. Our study identified four groups of factors influencing the uptake of digital health interventions, some of which were also highlighted in the existing literature.

First and foremost, having the aid of digital health or not, human interaction remains a critical ingredient to successful health service delivery, which emphasizes patient-supporter interaction as much as, if not more than, patient technology or provider technology interaction^22^. Second, the importance of user-centered design was repeatedly mentioned in the literature, which should entail user engagement in co-design, customization to personal needs and preferences, and feedback evaluations^22,87^. Our findings also agreed with existing studies in that more attention should be given to disadvantaged populations with limited capacity to use digital health tools due to technical illiteracy, and that adequate training and continuous incentives should be in place to ensure their sustainable uptake^88,89^. Another highly considerable facilitator to digital health uptake is the integration with existing health systems or local service delivery models, as was mentioned in two of the included studies in China^71,74^, which was also recommended by a WHO policy brief^90^. Finally, the optimal uptake of digital health interventions needs to be enhanced by political commitment for regulation, standardization, and support^91^. For future studies to optimally navigate the various factors influencing digital health uptake, we recommend the use of multiple methods such as qualitative research to pre-estimate local contexts and needs before implementation of digital health interventions.

This study has the strength of shifting the attention for digital health interventions from high-income countries and hospitals settings to the under-represented primary healthcare settings of LMICs. Our inclusion of both trials and reviews in the study provided insights for various contexts in LMICs. However, several limitations should also be acknowledged. First, we only focused on academic publications in English, and could not include grey literatures relating to digital health, especially in non-English speaking settings. We tried to mitigate this potential loss of information by including review papers that were published in English but investigated non-English publications in their analyses. Second, given the high level of heterogeneity in the included studies, we were not able to conduct a rigorous quantitative synthesis such as meta-analysis. Instead, we conducted a narrative synthesis with the available information, which sufficed to address our research questions and also pointed to future research directions to conduct more quantitative synthesis on specific types and elements of digital health interventions.

A blueprint for ideal digital health interventions should not only benefit individual health management and health facility service delivery, but also empower public health governance and policymaking through interoperable and reliable data services^77^. All these enhanced functionalities are needed, particularly in primary health care of LMICs. Our findings highlight both promises and limitations in the effectiveness of digital health interventions for NCD patients and health providers and suggest multiple factors to consider for industrial and governmental stakeholders in the initiation of digital health interventions, including political commitment, technology advancement, interactivity, integration with existing systems, and user-centered design and incentives. For future research, we call for more large-scale trials to further evaluate the real-world impact of digital health interventions in multiple aspects, particularly in the delivery of NCD services in primary health care of LMICs.

## Methods

### Search strategy and selection criteria

The following steps were guided by PRISMA guidelines (PRISMA-ScR, Supplementary Table 1)^92^. We searched PubMed, Embase, and Web of Science using three groups of keywords in the search syntax and required at least one keyword from each group: (1) digital health-related keywords, which included terminologies that describe subsets of digital health, such as “eHealth” (the use of information communication technologies for health^93^), “mHealth” (a subset of digital health enabled by mobile devices^94^), and specific digital health interventions, such as SMS and EHR; (2) NCD-related keywords, which included general terms such as “chronic conditions” and also specific conditions such as “hypertension” and “diabetes”; and (3) primary healthcare related keywords, which included synonyms of primary health care such as “basic health care”. Specific search syntax with subject headings were customized based on the requirements of each database, including Pubmed (using “title/abstract”), Embase (using “title/abstract/keywords”), and Web of Science (using “topic”). See Supplementary Notes 1 for the complete search syntax.

For inclusion criteria, we included all types of review papers, such as systematic reviews, scoping reviews, and umbrella reviews, and all types of experimental studies, including RCTs, cluster RCTs, and quasi-experimental studies, that (1) focused on the use of digital health interventions for NCD management, which entailed various healthcare activities, including prevention, treatment, and rehabilitation; (2) were conducted in primary healthcare settings; (3) were conducted in LMICs, which was based on World Bank’s classifications by income; and (4) were published from January 1, 2010, to ensure the timeliness of the findings. Of note, the search was completed on April 30, 2021. For exclusion criteria, we excluded: (1) studies that were conducted exclusively in high-income countries or in hospital settings; (2) study protocols, after attempting to find their completed publications; (3) papers of which the full-texts remained inaccessible (e.g., conference abstracts/proceedings) after contacting the corresponding author and seeking support from library staff; (4) qualitative evaluation studies of past trials, and (5) papers that were not written in English. Of note, we did not exclude studies that were conducted in both high-income countries and LMICs. We also did not include studies that focused on mental health—through an important NCD issue–because of the major distinctions between mental health and other NCDs with respect to their required resources and management models.

Trained researchers (S.X., H.L., E.D., A.N., R.D., and S.K.) conducted three rounds of screening, first by title, then by abstract, and finally by full-text, based on the inclusion and exclusion criteria. Reasons for exclusion were provided to studies excluded at the full-text screening stage. Each article was independently screened by at least two reviewers. Discrepancies between the reviewers were discussed in group meetings until an agreement was reached. We used EndNote X9 to manage the literature database.

### Data extraction and analysis

Trained reviewers (S.X., H.L., E.D., A.N., R.D., and S.K.) independently extracted the following data and compared their results for consistency. First, we extracted the basic information of each study, including the title, year of publication, author’s name, and study design. Then, in order to identify gaps in the use of digital health interventions for NCD management in primary health care of LMICs (Objective 1), we further extracted information about the specific digital health interventions that were covered by each study. Of note, the identified digital health interventions were not necessarily exclusively used for NCD management only, and some of them might also simultaneously contribute to other primary healthcare services (e.g., infectious disease management, maternal and child health services). To explore the effectiveness of the digital health interventions (Objective 2), we extracted information about the effectiveness of the digital health interventions according to the study outcomes, such as improvements in blood pressure control, patients’ behavioral changes, or users’ acceptance or satisfaction with health services. Finally, to identify factors influencing the uptake of the digital health interventions (Objective 3), we performed an inductive content analysis on the results, discussion, and/or implications sections of the included studies where applicable, to identify factors that influenced the uptake of the digital health interventions for NCD management at the primary healthcare level. Inductive content analysis was used because it enabled data-driven identification of themes (i.e., the “factors”) in a bottom-up manner from findings of the included studies.

We assessed the study quality of the included trials, following National Heart, Lung, and Blood Institute’s Study Quality Assessment Tools for the quality assessment^95^. The trials were categorized into good, fair, or suboptimal quality, considering their research practices including randomization, blinding, sample size sufficiency, pre-specification of outcomes, and intent-to-treat analysis.

### Study frameworks

**Fig. 4:**
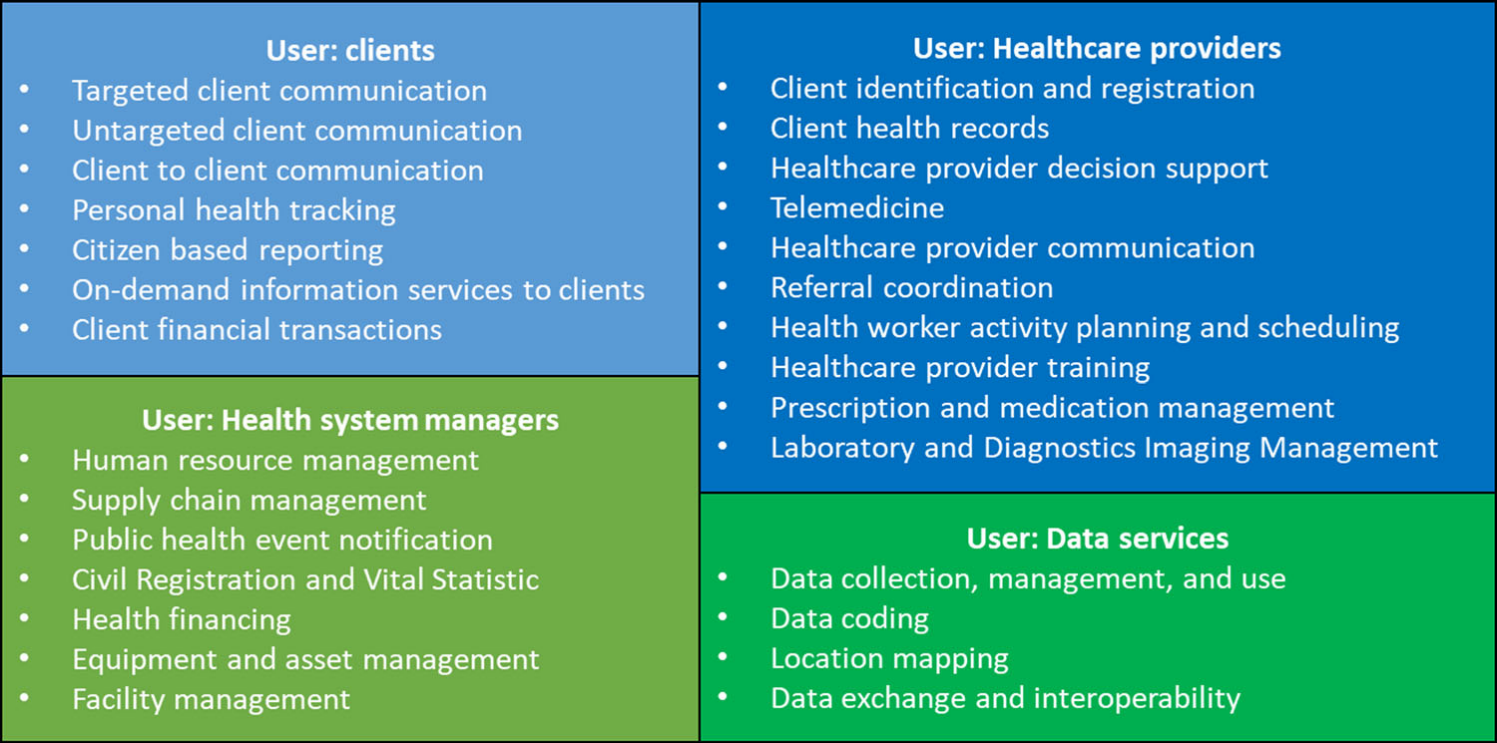
WHO classification of digital health interventions, adapted from WHO 2018^4^. The WHO classification categorized digital health interventions into four groups by different users: clients, healthcare providers, health system managers, and data services.

To identify gaps in the use of digital health interventions for NCD management in primary healthcare settings of LMICs, we utilized WHO’s Classification of digital health interventions as the guiding framework for benchmarking (Fig. 4)^4^. The WHO classification categorized digital health interventions into four groups by different users: (1) clients, which refers to potential or current users of health services—in our study, they are referred to as people with NCDs; (2) healthcare providers, which refers to the health workforce to deliver health services—in our study, they are referred to as primary healthcare providers; (3) health system managers, which refers to people involved in the administration and oversight of public health systems; and (4) data services, which refers to cross-cutting functionality for data collection, synthesis, use, and exchange^4^.

The health system quality of care framework provided six relevant dimensions that constitute the quality of health care^96^, and it was previously applied in a systematic review on mobile health tools for NCD management^97^. We applied these six dimensions to determine how the included studies attempted to use digital health interventions to improve NCD management in primary health care: (1) Effectiveness: being need-based, adherent to the evidence base, and resulting in improving health; (2) Efficiency: maximizing resource use and avoiding waste; (3) Accessibility: being timely, geographically reasonable, skillful, and resourceful; (4) Acceptability: considering individual preferences and aspirations as well as community cultures; (5) Equity: not varying in quality due to personal characteristics; and (6) Safety: minimizing risks and harm to service users^96^.

### Reporting summary

Further information on research design is available in the Nature Portfolio Reporting Summary linked to this article.

## Supplementary information

Supplementary information

Reporting Summary Checklist

## Data Availability

The data underlying this manuscript is based on existing publications and is available in the referenced literature or from the corresponding authors upon reasonable request.
