# Supplementary material for: Digital health interventions for non-communicable disease management in primary health care in low-and middle-income countries
Source: NPJ Digit Med. 2023 Feb 1;6:12. doi: 10.1038/s41746-023-00764-4 (PMC9889958; doi:10.1038/s41746-023-00764-4)
Supplement: Supplementary file 1 — Supplementary information [file 41746_2023_764_MOESM1_ESM.pdf]

Supplementary Table 1: Preferred reporting items for systematic reviews and meta-analyses extension for scoping reviews (PRISMA-ScR) checklist

SECTION	ITEM	PRISMA-ScR CHECKLIST ITEM	REPORTED ON PAGE #
**TITLE**			
Title	1	Identify the report as a scoping review.	Page 1
**ABSTRACT**			
Structured summary	2	Provide a structured summary that includes (as applicable): background, objectives, eligibility criteria, sources of evidence, charting methods, results, and conclusions that relate to the review questions and objectives.	Page 2
**INTRODUCTION**			
Rationale	3	Describe the rationale for the review in the context of what is already known. Explain why the review questions/objectives lend themselves to a scoping review approach.	Page 3
Objectives	4	Provide an explicit statement of the questions and objectives being addressed with reference to their key elements (e.g., population or participants, concepts, and context) or other relevant key elements used to conceptualize the review questions and/or objectives.	Page 3-4
**METHODS**			
Protocol and registration	5	Indicate whether a review protocol exists; state if and where it can be accessed (e.g., a Web address); and if available, provide registration information, including the registration number.	NA
Eligibility criteria	6	Specify characteristics of the sources of evidence used as eligibility criteria (e.g., years considered, language, and publication status), and provide a rationale.	Page 4
Information sources*	7	Describe all information sources in the search (e.g., databases with dates of coverage and contact with authors to identify additional sources), as well as the date the most recent search was executed.	Page 4
Search	8	Present the full electronic search strategy for at least 1 database, including any limits used, such that it could be repeated.	Page 4, Appendix 2
Selection of sources of evidence†	9	State the process for selecting sources of evidence (i.e., screening and eligibility) included in the scoping review.	Page 4
Data charting process‡	10	Describe the methods of charting data from the included sources of evidence (e.g., calibrated forms or forms that have been tested by the team before their use, and whether data charting was done independently or in duplicate) and any processes for obtaining and confirming data from investigators.	Page 5
Data items	11	List and define all variables for which data were sought and any assumptions and simplifications made.	Page 5
Critical appraisal of individual sources of evidence§	12	If done, provide a rationale for conducting a critical appraisal of included sources of evidence; describe the methods used and how this information was used in any data synthesis (if appropriate).	Page 5
Synthesis of results	13	Describe the methods of handling and summarizing the data that were charted.	Page 5-6
**RESULTS**			
Selection of sources of evidence	14	Give numbers of sources of evidence screened, assessed for eligibility, and included in the review, with reasons for exclusions at each stage, ideally using a flow diagram.	Page 7
Characteristics of sources of evidence	15	For each source of evidence, present characteristics for which data were charted and provide the citations.	Page 8
Critical appraisal within sources of evidence	16	If done, present data on critical appraisal of included sources of evidence (see item 12).	Page 8, Appendix 4
Results of individual sources of evidence	17	For each included source of evidence, present the relevant data that were charted that relate to the review questions and objectives.	Page 8-17, Appendix 3-4
Synthesis of results	18	Summarize and/or present the charting results as they relate to the review questions and objectives.	Page 8-17
**DISCUSSION**			
Summary of evidence	19	Summarize the main results (including an overview of concepts, themes, and types of evidence available), link to the review questions and objectives, and consider the relevance to key groups.	Page 18
Limitations	20	Discuss the limitations of the scoping review process.	Page 20
Conclusions	21	Provide a general interpretation of the results with respect to the review questions and objectives, as well as potential implications and/or next steps.	Page 21
**FUNDING**			
Funding	22	Describe sources of funding for the included sources of evidence, as well as sources of funding for the scoping review. Describe the role of the funders of the scoping review.	Page 21

JBI = Joanna Briggs Institute; PRISMA-ScR = Preferred Reporting Items for Systematic reviews and Meta-Analyses extension for Scoping Reviews.

* Where *sources of evidence* (see second footnote) are compiled from, such as bibliographic databases, social media platforms, and Web sites.

† A more inclusive/heterogeneous term used to account for the different types of evidence or data sources (e.g., quantitative and/or qualitative research, expert opinion, and policy documents) that may be eligible in a scoping review as opposed to only studies. This is not to be confused with *information sources* (see first footnote).

‡ The frameworks by Arksey and O'Malley (6) and Levac and colleagues (7) and the JBI guidance (4, 5) refer to the process of data extraction in a scoping review as data charting.

§ The process of systematically examining research evidence to assess its validity, results, and relevance before using it to inform a decision. This term is used for items 12 and 19 instead of "risk of bias" (which is more applicable to systematic reviews of interventions) to include and acknowledge the various sources of evidence that may be used in a scoping review (e.g., quantitative and/or qualitative research, expert opinion, and policy document).

From: Tricco AC, Lillie E, Zarin W, O'Brien KK, Colquhoun H, Levac D, et al. PRISMA Extension for Scoping Reviews (PRISMAScR): Checklist and Explanation. Ann Intern Med. 2018;169:467–473. doi: 10.7326/M18-0850.

Supplementary Notes 1: Search syntax on Pubmed, Embase, and Web of Science

Note: specific search syntax with subject headings were customized based on requirements of each database, including Pubmed (using “title/abstract”), Embase (using “title/abstract/keywords”), and Web of Science (using “topic”).

Group A-Digital health related:

"digital health" OR "dhealth" OR "d-health" OR "ehealth" OR "electronic health" OR "e-health" OR "mhealth" OR "mobile health" OR "m-health" OR "telehealth" OR "teletherapy" OR "tele-therapy" OR "tele-intervention" OR "teleintervention" OR "telecare" or "tele-care" OR "EHR" OR "electronic health record" OR "electronic medical record" OR "EMR" OR "blood pressure monitoring" OR "bp monitoring" OR "information communication technology" OR "ICT" OR "short-message service" OR "sms" OR "smart phone" OR "cell phone" OR "cell-phone" OR "feature phone" OR "mobile phone" OR "telephone" OR "app" OR "wireless health" or "healthcare technology" OR "medical information system" OR "telemonitoring" OR "tele-monitoring" OR "telepresence" OR "tele-presence" OR "electronic health information" OR "teleconsultation" OR "tele-consultation" OR "e-rehabilitation" OR "personal digital assistant" OR "text-message"

AND

Group B-Non-communicable disease related:

"hypertension" OR "HTN" OR "blood pressure" OR "diabetes" OR "heart disease" OR "stroke" OR "cerebrovascular disease" OR "CVD" OR "cardiovascular disease" OR "non-communicable" OR "NCD" OR "chronic disease" OR "non-infectious disease" OR "cardiometabolic disease" OR "CMD" OR "cardiometabolic condition" OR "cardiometabolic patient" OR "metabolic syndrome"

AND

Group C-Primary health care related:

"PHC" OR "primary health care" OR "primary healthcare" OR "primary care" OR "primary health" OR "basic healthcare" OR "essential healthcare" OR "healthcare at the grassroot level"

Time frame: 2010.01.01~2021.04.30

Supplementary Table 2: Data extraction on basic characteristics of the 31 included reviews

First Author, Year	Name of publication	Review type	Number of reviewed studies	Types of reviewed studies	Range of sample size of reviewed studies
Agarwal R 2011	Role of Home Blood Pressure Monitoring in Overcoming Therapeutic Inertia and Improving Hypertension Control A Systematic Review and Meta-Analysis	Systematic review (with meta-analysis)	37	Randomized controlled trials (RCTs)	15-1,325
de Jongh T 2012	Mobile phone messaging for facilitating self-management of long-term illnesses	Systematic review	6	RCTs	16-67
Baron J 2012	The impact of mobile monitoring technologies on glycosylated hemoglobin in diabetes: a systematic review	Systematic review	20	RCTs, single-group pre and post	10-328
Mair FS 2012	Factors that promote or inhibit the implementation of e-health systems: an explanatory systematic review	Umbrella review	37	Systematic reviews, narrative reviews, qualitative meta-syntheses or meta-ethnographies	NA
Buhi ER 2013	Mobile phone-based behavioural interventions for health: A systematic review	Systematic review	34	RCTs, quasi-experiment studies	10-1,705
El-Gayar O 2013	A systematic review of IT for diabetes self-management: Are we there yet?	Systematic review	104	RCTs, cluster-RCT	6-2,924
Bloomfield GS 2014	Mobile health for non-communicable diseases in Sub-Saharan Africa: a systematic review of the literature and strategic framework for research	Systematic review	5	Chart review, single center report, prospective study, uncontrolled trials	22-1,160
Nhavoto JA 2014	Mobile technologies and geographic information systems to improve health care systems: a literature review	Non-specified review type	271	Peer-reviewed papers and conference proceedings with no specified types	5-5,800
Zapata BC 2015	Empirical studies on usability of mHealth apps: a systematic literature review	Systematic review	22	Empirical studies	NA
Beratarrechea A 2016	Use of m-Health Technology for Preventive Interventions to Tackle Cardiometabolic Conditions and Other Non-Communicable Diseases in Latin America- Challenges and Opportunities	Systematic review	9	RCTs, cluster-RCT, quasi-experimental (pre-post) studies, quasi-experimental and qualitative study	172-1,950
Beishuizen CRL 2016	Web-Based Interventions Targeting Cardiovascular Risk Factors in Middle-Aged and Older People: A Systematic Review and Meta-Analysis	Systematic review (with meta-analysis)	57	RCTs	61-2,140
Hsu J 2016	The top Chinese mobile health apps: a systematic investigation	Non-specified review type	234	Mobile phone applications	NA
Hou C 2016	Do mobile phone applications improve glycemic control (HbA1c) in the self-management of diabetes? A systematic review, meta-analysis, and grade of 14 randomized trials	Systematic review (with meta-analysis)	14	RCTs	7-67
Lewis J 2016	Recent worldwide developments in eHealth and mHealth to more effectively manage cancer and other chronic diseases–a systematic review	Umbrella review	15	Systematic reviews, narrative reviews	10-5,800
Cajita MI 2016	A systematic review of mHealth-based heart failure interventions	Systematic review	10	RCTs, quasi-experimental studies	6-710
Müller AM 2016	The effectiveness of e-& mHealth interventions to promote physical activity and healthy diets in developing countries: a systematic review	Systematic review	16	RCTs, quasi-experimental trials	22-69,219

Kebede MM 2017	Characterizing active ingredients of eHealth interventions targeting persons with poorly controlled type 2 diabetes mellitus using the behavior change techniques taxonomy: scoping review	Scoping review	32	RCTs	18-379
Miller L 2017	Mobile technology interventions for asthma self-management: systematic review and meta-analysis	Systematic review (with meta-analysis)	11	RCTs	16-288
Njoroge M 2017	Assessing the feasibility of eHealth and mHealth: a systematic review and analysis of initiatives implemented in Kenya	Systematic review	69	RCTs, quasi-experimental studies, cross-sectional studies, cohort studies, qualitative studies	NA
Wang Y 2017	A systematic review of application and effectiveness of mHealth interventions for obesity and diabetes treatment and self-management	Systematic review	24	RCTs, quasi-experiments	15-124
Kim BY 2017	Smart devices for older adults managing chronic disease: a scoping review	Scoping review	51	Pre-experimental design studies, RCTs, qualitative studies, quasi-experimental studies, mixed methods studies	4-471
Watkins JA 2018	mHealth text and voice communication for monitoring people with chronic diseases in low-resource settings: a realist review	Systematic review	4	RCTs	162-1,188
Byambasuren O 2018	Prescribable mHealth apps identified from an overview of systematic reviews	Umbrella review	6	Systematic reviews	17-1,929
Marcolino MS 2018	The impact of mHealth interventions: systematic review of systematic reviews	Umbrella review	23	Systematic reviews	9-107
Lu X 2019	Interactive mobile health intervention and blood pressure management in adults: a meta-analysis of randomized controlled trials	Systematic review (with meta-analysis)	11	RCTs	NA
Dounavi K 2019	Mobile health applications in weight management: a systematic literature review	Systematic review	39	RCTs, Non-randomized trials	NA
Almutairi N 2020	The effectiveness of patient activation intervention on type 2 diabetes mellitus glycemic control and self-management behaviors: A systematic review of RCTs	Systematic review	10	RCTs	120-711
Godinho MA 2020	mHealth for integrated people-centred health services in the Western Pacific: a systematic review	Systematic review	39	RCTs, cross-sectional studies, qualitative studies, quasi-experiments, diagnostic test accuracy studies	6-7,421
Gonçalves-Bradley DC 2020	Mobile technologies to support healthcare provider to healthcare provider communication and management of care	Systematic review (with meta-analysis)	19	Randomized trials, conference abstracts	85-5423
McNatt ZZ 2020	McNatt ZZ. Addressing noncommunicable diseases among urban refugees in the Middle East and North Africa-a scoping review	Scoping review	11	Policy evaluation, mixed-method studies, cohort studies, descriptive study	NA
Pai RR 2021	Bibliometric analysis and methodological review of mobile health services and applications in India	Systematic review	158	Not specified	NA

Supplementary Table 3: Data extraction on basic characteristics of the 21 included trials

First Author, Year	Name of publication	Trial type	Study country	Disease types	Sample size	Quality assessment
dos Santos MVR 2013	A Telehealth Strategy for Increasing Adherence in the Treatment of Hypertension in Primary Care	Quasi-experiment (pre-post)	Brazil	Hypertension	502 patients and 21 doctors	Sub-optimal
Tian M 2015	A cluster-randomized, controlled trial of a simplified multifaceted management program for individuals at high cardiovascular risk (SimCard trial) in rural Tibet, China, and Haryana, India	Cluster-randomized controlled trial (Cluster-RCT)	China, India	People with high cardiovascular risks	2,086	Good
Bobrow K 2016	Mobile Phone Text Messages to Support Treatment Adherence in Adults With High Blood Pressure (SMS-Text Adherence Support [StAR]): A Single-Blind, Randomized Trial	Randomized controlled trial (RCT)	South Africa	Hypertension	1,372	Good
Ajay VS 2016	Development of a Smartphone-Enabled Hypertension and Diabetes Mellitus Management Package to Facilitate Evidence-Based Care Delivery in Primary Healthcare Facilities in India: The mPower Heart Project	Quasi-experiment (pre-post)	India	Hypertension and diabetes	6,979	Sub-optimal
Fernandes BSM 2016	Evaluation of the telephone intervention in the promotion of diabetes self-care: a randomized clinical trial	RCT	Brazil	Diabetes	210	Good
Maia JX 2016	The impact of a clinical decision support system in diabetes primary care patients in a developing country	Quasi-experiment (pre-post)	Brazil	Diabetes	145	Sub-optimal
Diehl LA 2017	InsuOnline, an Electronic Game for Medical Education on Insulin Therapy: A Randomized Controlled Trial With Primary Care Physicians	RCT	Brazil	Diabetes	134 doctors	Good
Varleta P 2017	Mobile phone text messaging improves antihypertensive drug adherence in the community	RCT	Chile	Hypertension	314	Fair
Kleczka B 2018	Rubber stamp templates for improving clinical documentation: A paper-based, m-Health approach for quality improvement in low-resource settings	Quasi-experiment (pre-post)	Kenya	Hypertension, diabetes, chronic respiratory diseases	70	Sub-optimal
Nascimento BR 2018	Integration of echocardiographic screening by non-physicians with remote reading in primary care	Feasibility/pilot study	Brazil	Asymptomatic patients referred for clinical indication	1,004	Sub-optimal
Visanuyothin S 2018	An integrated program with home blood-pressure monitoring and village health volunteers for treating poorly controlled hypertension at the primary care level in an urban community of Thailand	Quasi-experiment	Thailand	Hypertension	122	Sub-optimal
Beratarrechea A 2019	Using mHealth Tools to Improve Access and Coverage of People With Public Health Insurance and High Cardiovascular Disease Risk in Argentina: A Pragmatic Cluster Randomized Trial	Cluster-RCT	Argentina	Diabetes mellitus, hypertension, hyperocholestorelemia	755	Good
Menezes P 2019	Use of a Mobile Phone App to Treat Depression Comorbid With Hypertension or Diabetes: A Pilot Study in Brazil and Peru	Feasibility/pilot study	Brazil	Hypertension, diabetes, with depressive symptoms	66	Sub-optimal

Pichayapinyo P 2019	Feasibility study of automated interactive voice response telephone calls with community health nurse follow-up to improve glycaemic control in patients with type 2 diabetes	Quasi-experiment (pre-post)	Thailand	Type-2 diabetes	35 patients and 6 nurses	Sub-optimal
Prabhakaran D 2019	Effectiveness of an mHealth-Based Electronic Decision Support System for Integrated Management of Chronic Conditions in Primary Care: The mWellcare Cluster-Randomized Controlled Trial	Cluster-RCT	India	Hypertension, type-2 diabetes	3,698	Good
Silveira DV 2019	Development and Evaluation of a Mobile Decision Support System for Hypertension Management in the Primary Care Setting in Brazil: Mixed-Methods Field Study on Usability, Feasibility, and Utility	Feasibility/pilot study	Brazil	Hypertension	10 family physicians	Sub-optimal
Debon R 2020	Effects of using a mobile health application on the health conditions of patients with arterial hypertension: A pilot trial in the context of Brazil's Family Health Strategy	Feasibility/pilot study	Brazil	Hypertension	39	Sub-optimal
Güner T. A. 2020	The effect of diabetes education and short message service reminders on metabolic control and disease management in patients with type 2 diabetes mellitus	Quasi-experiment (pre-post)	Turkey	Type-2 diabetes	101	Sub-optimal
Lee JY 2020	Telemonitoring and Team-Based Management of Glycemic Control on People with Type 2 Diabetes: a Cluster-Randomized Controlled Trial	Cluster-RCT	Malaysia	Type-2 diabetes	240	Good
Montrivade S 2020	Hypertension Subtypes among Thai Hypertensives: An Analysis of Telehealth-Assisted Instrument in Home Blood Pressure Monitoring Nationwide Pilot Project	Feasibility/pilot study	Thailand	Hypertension	1,184	Sub-optimal
Yan LL 2021	Effectiveness of a primary care-based integrated mobile health intervention for stroke management in rural China (SINEMA): A cluster-randomized controlled trial	Cluster-RCT	China	Stroke	1,250	Good
